# Controlling Dzyaloshinskii-Moriya Interaction via Chirality Dependent Atomic-Layer Stacking, Insulator Capping and Electric Field

**DOI:** 10.1038/s41598-018-30063-y

**Published:** 2018-08-17

**Authors:** Hongxin Yang, Olivier Boulle, Vincent Cros, Albert Fert, Mairbek Chshiev

**Affiliations:** 1 Univ. Grenoble Alpes, CEA, CNRS, Grenoble INP, INAC-Spintec, 38000 Grenoble, France; 2 Unité Mixte de Physique CNRS, Thales, University Paris-Sud, University Paris-Saclay, 91767 Palaiseau, France; 3 Key Laboratory of Magnetic Materials and Devices, Ningbo Institute of Materials Technology and Engineering, Chinese Academy of Sciences, Ningbo, 315201 China

## Abstract

Using first-principles calculations, we demonstrate several approaches to control Dzyaloshinskii-Moriya Interaction (DMI) in ultrathin films with perpendicular magnetic anisotropy. First, we find that DMI is significantly enhanced when the ferromagnetic (FM) layer is sandwiched between nonmagnetic (NM) layers inducing additive DMI in NM1/FM/NM2 structures. For instance, when two NM layers are chosen to induce DMI of opposite chirality in Co, e.g. NM1 representing Au, Ir, Al or Pb, and NM2 being Pt, the resulting DMI in NM1/Co/Pt trilayers is enhanced compared to Co/Pt bilayers. Moreover, DMI can be significantly enhanced further in case of using FM layer comprising Fe and Co layers. Namely, it is found that the DMI in Ir/Fe/Co/Pt structure can be enhanced by 80% compared to that of Co/Pt bilayers reaching a very large DMI amplitude of 5.59 meV/atom. Our second approach for enhancing DMI is to use oxide capping layer. We show that DMI is enhanced by 60% in Oxide/Co/Pt structures compared to Co/Pt bilayers. Moreover, we unveiled the DMI mechanism at Oxide/Co interface due to Rashba effect, which is different to Fert-Levy DMI at FM/NM interfaces. Finally, we demonstrate that DMI amplitude can be modulated using an electric field with an efficiency factor comparable to that of the electric field control of perpendicular magnetic anisotropy in transition metal/oxide interfaces. These approaches of DMI controlling pave the way for skyrmion and domain wall motion-based spintronic applications.

## Introduction

The possibility to manipulate magnetization at nanoscale using the coupling between electron’s spin and its motion (orbital angular momentum) has led to the emergence of a new research field named “spin-orbitronics”. A fascinating example of the impact of the spin-orbit coupling (SOC) on the magnetization profile is the chiral spiral or skyrmion magnetic orders observed at the surface of magnetic ultrathin films^1–10^. Such spin configurations are driven by an additional term in the exchange interaction, namely Dzyaloshinskii-Moryia interaction (DMI)^11–21^, which arises from the presence of SOC and inversion symmetry breaking^21,22^. Novel out-of-equilibrium spin transport phenomena also result in such structure, such as the spin-orbit torques (SOT) exerted on the magnetization when injecting a current, leading to fast current induced domain wall motion and magnetization reversal^23–27^. This has led to new concept of magnetic memory, such as skyrmion-based memory, where the information is coded by nm scale magnetic skyrmions in nanotracks and manipulated using current pulses^22,28,29^. However, for the applications, there are still many issues to solve, for example in order to increase the stability of the chiral dependent domain walls (DW), a large DMI amplitude is critical. Furthermore, the maximum velocity of domain wall motion is also shown to strongly depend on DMI amplitude. Therefore, the search for material stack with a large DMI is the key to realize stable skyrmions and chiral DW for spin-orbitronic devices.

**Figure 1:**
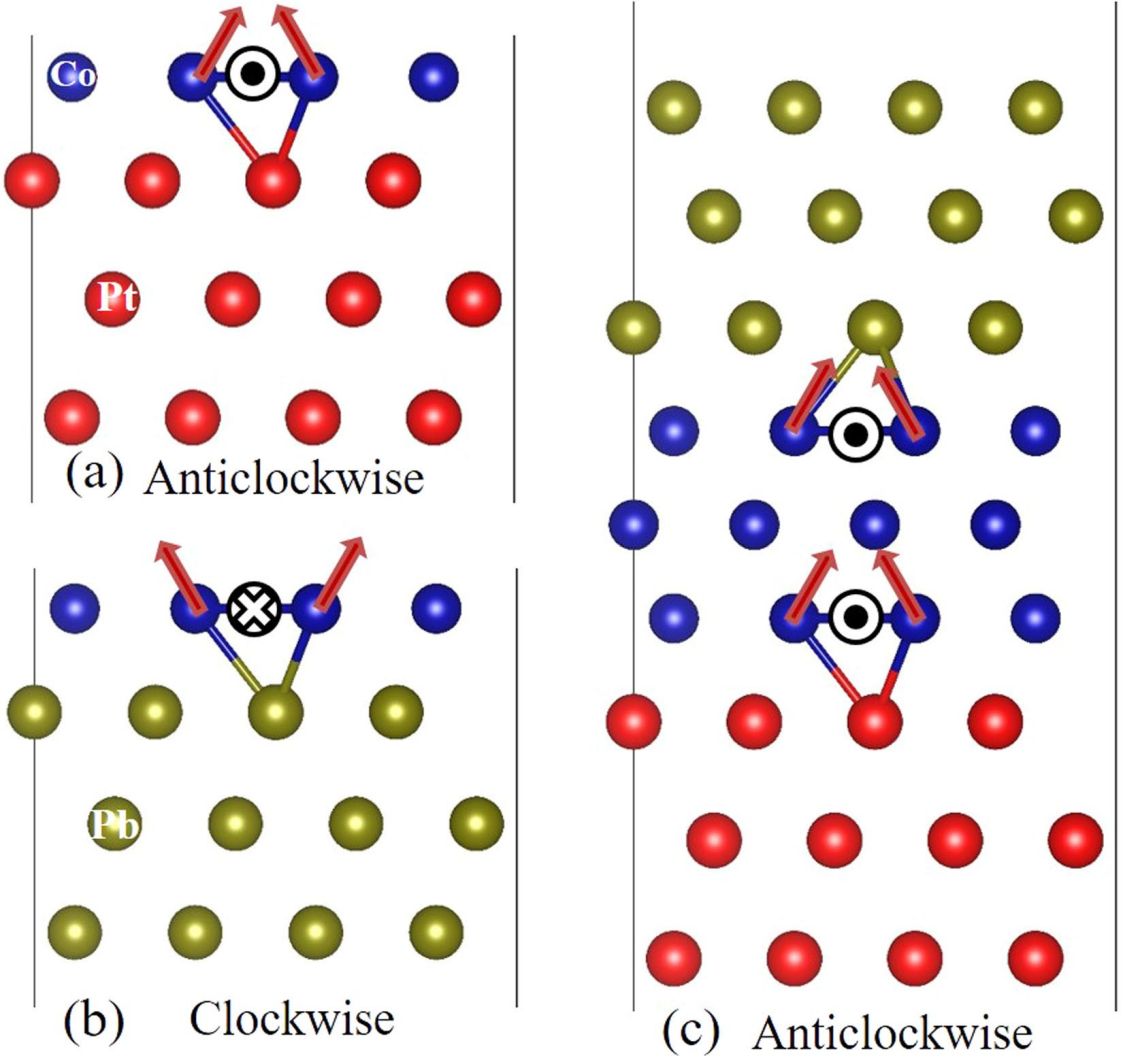
Schematic structures with anticlockwise DMI in Co/Pt bilayers (**a**), clockwise DMI in Co/Pb bilayers (**b**) and enhanced DMI in Pb/Co/Pt trilayers (**c**).

In this Article, we propose several approaches to enhance DMI in ultrathin magnetic films. First, we show that DMI can be magnified via multilayer stacking of FM and NM metals when the ferromagnetic (FM) layer is sandwiched between nonmagnetic (NM) layers inducing additive DMI in NM1/FM/NM2 structures^16,30–32^. Here, in case of DMI enhancement, the key is to find required DMI chiralities for additive effects at successive interfaces. For example, in asymmetric trilayers of Pb/Co/Pt, where the DMI chirality at separated Co/Pt and Co/Pb interfaces is opposite as shown in Fig. 1(a,b), respectively. Due to the inversion geometry stacking from Co/Pb to Pb/Co in forming Pb/Co/Pt trilayers, the sign of DMI at the interface of Pb/Co is reversed resulting in an overall enhanced anticlockwise DMI as schematiclly shown in Fig. 1(c). Another approach we propose is to use a capping oxidized layer, such as MgO, on top of Co/Pt bilayers, which is shown to efficiently enhance the DMI. Here, the DMI enhancement mechanism arising from MgO/Co interface is found due to Rashba effect. Finally, we demonstrate that DMI can be efficiently tailored by applying an electric field (EF). This unveils the possibility to control DMI and perpendicular magnetic anisotropy (PMA) simultaneously via electric field which opens an efficient route towards EF-manipulation of magnetic skyrmions.

## Methods

To calculate DMI, we employ the same approach as in our previous work^33^ based on the Vienna *ab initio* simulation package (VASP)^34,35^, which represents an adaptation to the case of layered structures of the method used for DMI calculations in bulk frustrated systems and insulating chiral-lattice magnets^36,37^. The electron-core interactions are described by the projected augmented wave (PAW) method for the pseudopotentials with exchange correlation energy calculated within the generalized gradient approximation (GGA) of the Perdew-Burke-Ernzerhof (PBE) form^38^. The cutoff energies for the plane wave basis set used to expand the Kohn-Sham orbitals were chosen to be 320 eV and 500 eV for structures comprising only metals and those with oxygen, respectively. The Monkhorst-Pack scheme was used for the Γ-centered 4 × 16 × 1 k-point sampling which is enough to ensure the convergence.

Our DMI strength parameter *d* is derived from the energy difference between clockwise and anticlockwise magnetic spiral configurations by identifying this difference with what can be expected from an analytical expression of pair DMIs for the atoms in a single Co atomic layer^33^. This *d* given in eV per atom can be seen as the coefficient of pair DMIs concentrated in a single atomic layer and producing an equivalent effect^33^. In other words the microscopic DMI constant *d* represents a property of the interface and is independent of *q* value. The calculation of *d* has been performed in three steps. First, structural relaxations were performed until the forces become smaller than 0.001 eV/Å for determining the most stable interfacial geometries. Next, the Kohn-Sham equations were solved, with no SOC, to find out the charge distribution of the system’s ground state. Finally, SOC was included and the self-consistent total energy of the system was determined as a function of the orientation of the magnetic moments which were controlled by using the constrained method implemented in VASP^34,35^.

## Results and Discussion

### DMI Enhancement via Chirality Dependent Atomic-Layer Stacking

**Figure 2:**
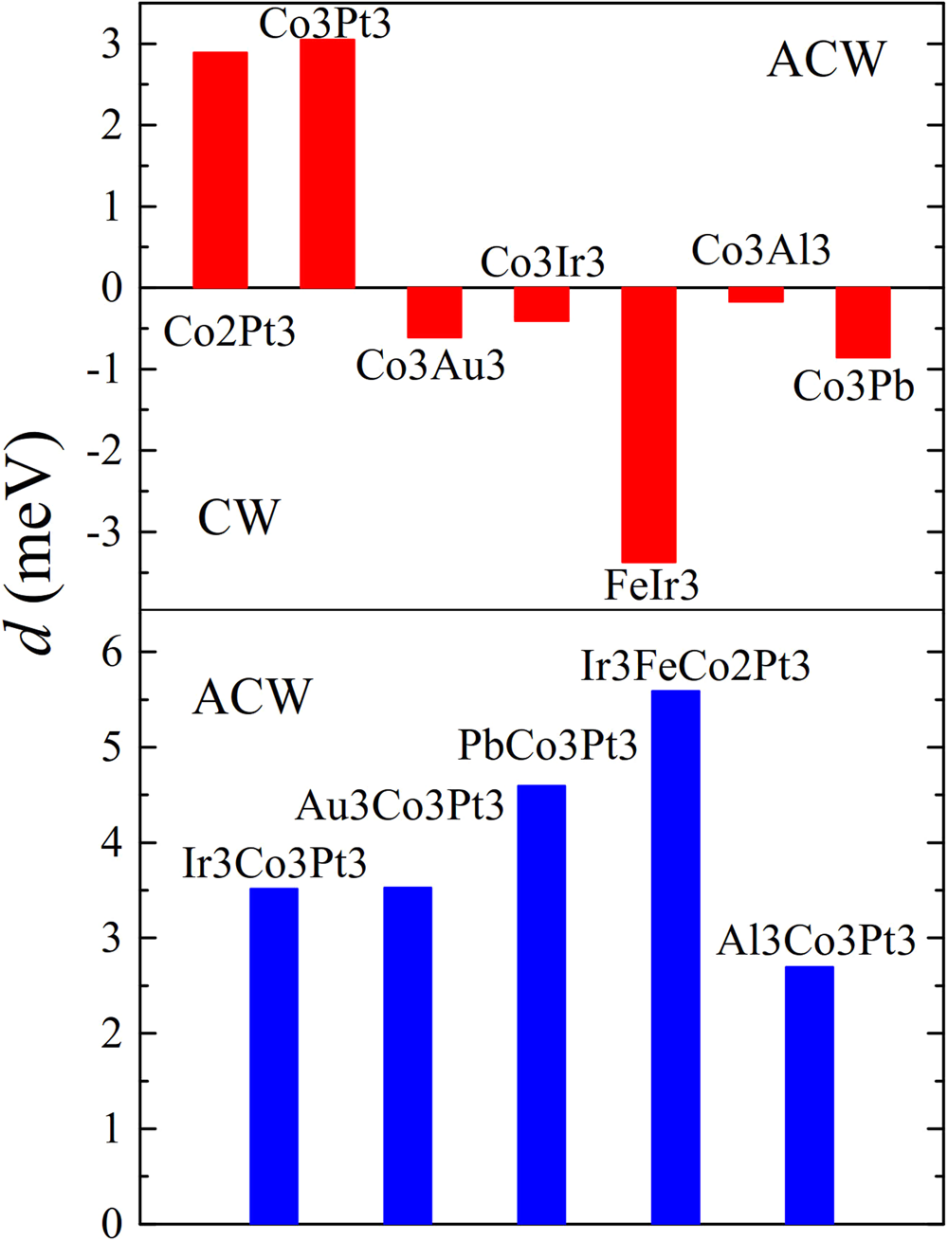
Calculated microscopic DMI, *d*, for bilayer structures (upper panel), and trilayers (lower panel).

In order to design metallic trilayers with efficiently enhanced DMI, we first systematically investigated DMI in FM/NM bilayers. The calculated results are summarized in the upper panel of Fig. 2. One can see that DMI chirality is anticlockwise (ACW) for Co/Pt bilayers while for Co on Au, Ir, Pb and Al substrates, DMI shows a clockwise (CW) chirality. We note that the DMI magnitude is larger for Fe on top of Ir compared to that of Co on Ir case. Let us now combine two interfaces of opposite DMI chiralities, e.g. Co/Pt and Co/Pb shown in Fig. 1(a,b), with reverse stacking of the second interface so that both interfaces in the resulting Pb/Co/Pt structure (Fig. 1(c)) possess ACW chirality. As summarized in the bottom panel of Fig. 2, the resulting DMI strength for NM/Co/Pt trilayers is approximately equal to the sum of DMI magnitudes of the two interfaces, i.e. Co/Pt and Co/NM, constituting the trilayer structure.

Due to small DMI value at Co/NM interfaces in cases when NM is Al, Au or Ir the DMI strength in NM/Co/Pt trilayers is moderately enhanced (<15%) compared to that of Co/Pt bilayer, 3.05 meV/atom. However, in the case of Pb/Co/Pt trilayer, the DMI enhancement can reach up to 4.59 meV/atom. This is due to the high DMI value at the Pb/Co interface (0.85 meV/atom with CW chirality), as compared to that of Co/Au and Co/Ir interfaces. It is interesting to note that the total DMI of Pb/Co/Pt structure, 4.59 meV/atom, is larger than the sum of the DMI contributions from Co/Pt and Pb/Co bilayers, i.e. 3.05 meV/atom +0.85 meV/atom = 3.90 meV/atom. This discrepancy is due to the DMI from 2nd and 3rd interface layers^33^, and the influence of the electronic structure, especially the shifting of Fermi level in trilayers compared to the corresponding bilayers. This deviation is also observed in case of other trilayers (Fig. 2).

Next, we consider a four layer Ir/Fe/Co/Pt structure which can be seen as replacing one monolayer of Co in Ir/Co/Pt trilayer by Fe. This is particularly interesting since a single monolayer of Fe on Ir(111) possesses very large DMI compared to that of Co on Ir (Fig. 2). The calculated DMI for this structure is shown in the bottom panel of Fig. 2. One can see that there is a significant enhancement for DMI in Ir/Fe/Co/Pt compared to that in Ir/Co/Pt with amplitude up to 5.59 meV/atom which is 1.8 times larger compared to that of Co/Pt.

**Figure 3:**
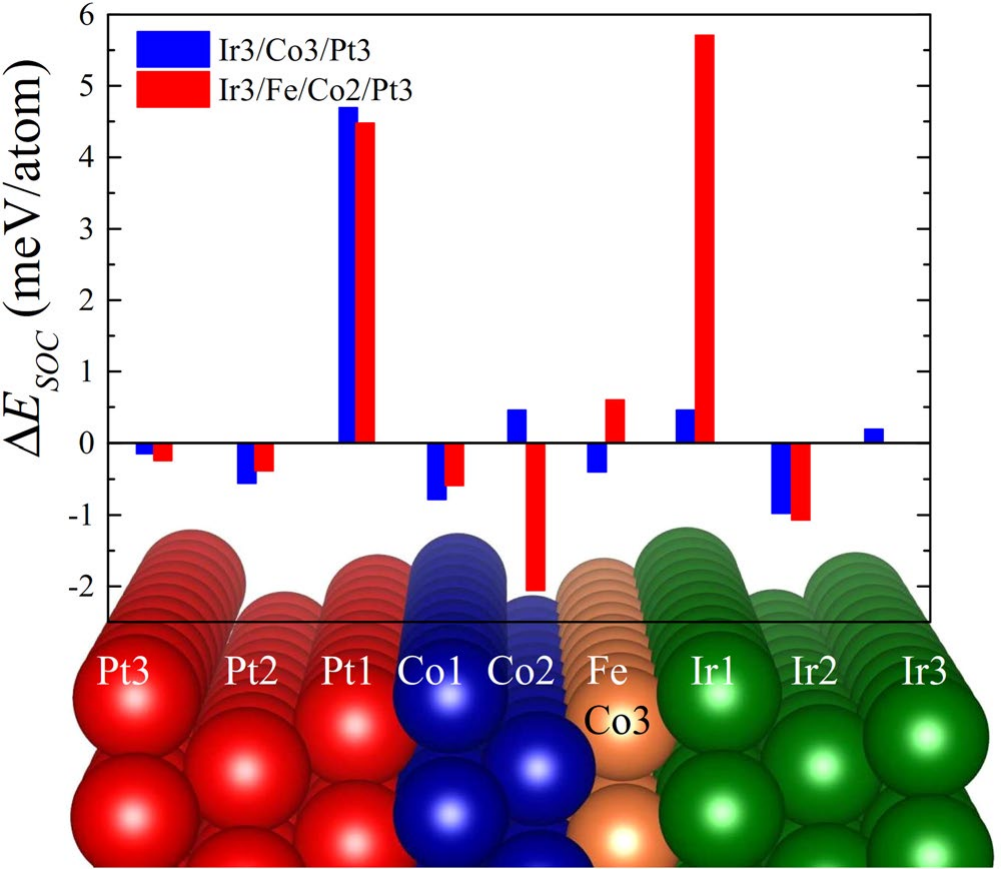
Layer-resolved distribution of the energy difference between clockwise and anticlockwise chiralities for Ir3Co3Pt3 (blue) and Ir3FeCo2Pt3 (red) structures, respectively.

In order to elucidate microscopic mechanisms of Fe induced DMI enhancement in this structure, we calculated the chirality-dependent SOC energy difference, Δ*E*_*SOC*_, which defines the DMI at metallic NM/FM interfaces according to Fert-Levy picture^21,33^, for both Ir3/Co3/Pt3 and Ir3/Fe/Co2/Pt3 as shown in Fig. 3. For the FM layer containing only three ML of Co (blue bars), the dominant SOC energy source Δ*E*_*SOC*_ is located at the first interfacial Pt layer, indicating large DMI in adjacent ferromagnetic Co1 layer^33^. At the same time, the Δ*E*_*SOC*_ at the Ir/Co interface is much smaller compared to that at the Co/Pt interface. However, when the adjacent Co to Ir layer is replaced by Fe, Δ*E*_*SOC*_ located at interfacial Ir layer is significantly increased (red bars) indicating a large DMI at Fe layer. This increase is attributed to the relative position of 5d states of NM in respect to the Fermi level which directly affects the SOC energy at the adjacent NM atoms^33,39^. Thus, Ir/Fe/Co/Pt structure is much more efficient than Ir/Co/Pt for providing high DMI values. Note that during the long submission period of this article, experimental group has confirmed our prediction^40^.

### DMI Enhancement via Insulator Capping

**Figure 4:**
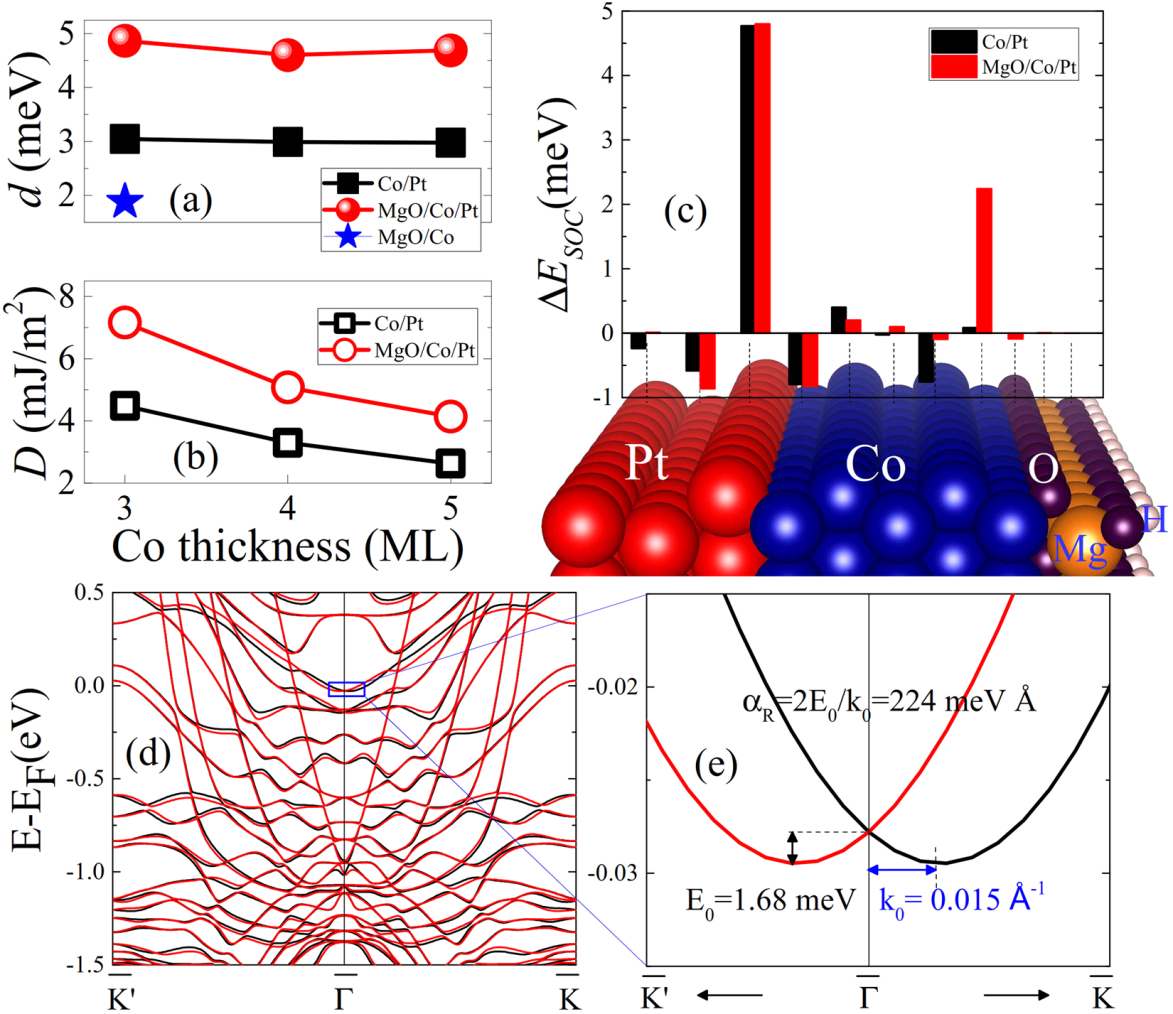
(**a**) *Ab* *initio* calculated DMI for microscopic, *d*, in Co/Pt (solid black squires) and MgO/Co/Pt (solid red balls) and MgO/Co (blue star) structures as a function of Co thickness. (**b**) *Ab* *initio* calculated micromagnetic DMI, *D*, in Co/Pt (empty black squires) and MgO/Co/Pt (empty red circles) as a function Co thickness. (**c**) spin-orbit coupling energy difference, Δ*E*_*SOC*_, in Co/Pt (black bars) and MgO/Co/Pt (red bars) between clockwise and anticlockwise spin spirals. (**d**) Band structure for MgO capped 3 ML of Co when the magnetization is setting along $\langle 110\rangle$ (black) and $\langle \bar{1}\bar{1}0\rangle$ (red) when SOC is switched on. (**e**) Zoom in of the band structure around $\bar{{\rm{\Gamma }}}$ point to calculate Rashba coefficient.

Another approach to enhance DMI is to add an insulator (I) capping layer on top of FM/NM to form I/FM/NM structure. These structures, notably AlOx/Co/Pt, MgO/Co/Pt or MgO/FeCo/Ta, are of major interest for the manipulation of magnetization^10,15,23–26,41^ in domain walls, skyrmions or nanomagnets via spin-orbit torques, where DMI is known to play an essential role in the magnetization reversal^27,42–44^. We consider the MgO/Co/Pt structure comprises 3 ML of Pt, 3 to 5 ML of Co, and MgO with surface passivated by hydrogen as shown in Fig. 4(c). The results show that DMI in MgO/Co/Pt is much larger, about 1.6 times, compared to that of Co/Pt bilayers for all the Co thicknesses considered. Furthermore, when varying the thickness of Co layer from 3–5 MLs in both Co/Pt and MgO/Co/Pt structures, the microscopic DMI *d* is not affected much suggesting that the Co/MgO interface has a similar impact on the electronic structure of the Co layer up to at least 5 monolayers (shown in Fig. 4(a)). Note, however, that the micromagnetic DMI constant *D* defined according to Eq. 4 in ref.^33^ is expected to decrease with increasing Co thickness as shown in Fig. 4(b). Recently, a large value of the DMI of 2 mJ/m^2^ has been measured using Brillouin Light Scattering experiments in sputtered ultrathin Pt/Co(1 nm)/MgO multilayer corresponding to 5 ML of Co, which is among the largest value measured so far in ultrathin sputtered films. Our simulations predict a large value of 3.9 mJ/m^2^ for this system. The lower experimental value can be explained by the disordered interface and grain structure in the case of sputtered thin films. In order to understand the mechanism of the DMI enhancement due to the MgO capping, we first calculated the DMI at MgO/Co interface and found the DMI amplitude about 1.88 meV/atom (Fig. 4(a)). Next, we performed the comparative analysis of SOC energy difference between CW and ACW spin spirals for Co/Pt [black bars] and MgO/Co/Pt [red bars] shown in Fig. 4(c). Very interestingly, one can see that SOC energy source for DMI at Co/Pt interface is located within the interfacial Pt layer, in agreement with Fert-Levy mechanism for DMI at metallic interfaces^33^. At MgO/Co interface, however, both the DMI and its SOC energy source are localized within the interfacial Co layer indicating a different mechanism. Recently, it was proposed that the Rashba spin-orbit coupling at the interface between a ferromagnet and an oxide can lead to DMI. This additional interaction can be explained as induced by the Rashba effect^45–47^ and can be expressed as *d* = 2 *k*_*R*_*A* where *A* indicates the exchange stiffness, ${k}_{R}=\frac{2{\alpha }_{R}{m}_{e}}{{\hslash }^{2}}$ is determined by the Rashba coefficient, *α*_*R*_, and effective mass, *m*_*e*_. The effective mass in Co was measured to be about 0.45 *m*_0_^48^ and the exchange stiffness, *A,* could be between 15.5 and 30 × 10^−12^ J/m^10,16,49^. The Rashba coefficient, *α*_*R*_, can then be extracted from *α*_*R*_ = 2E_0_/k_0_, where E_0_ is the Rashba splitting at the wave vector k_0_. We calculated the Rashba splitting for the MgO capped 3 ML of Co case by switching on SOC and putting the magnetization along $\langle 110\rangle$ and $\langle \bar{1}\bar{1}0\rangle$^50^, as shown in Fig. 4(d,e) with black and red curves, respectively. We used the band around Fermi level at the $\bar{{\rm{\Gamma }}}$ point, as shown in Fig. 4(e), to estimate the Rashba-type DMI, where the splitting, E_0_, is about 1.68 meV at k_0_ = 0.015 Å^−1^, which leads to a Rashba coefficient, *α*_*R*_, of 224 meV Å. Using this value, one obtains k_*R*_ = 26.6 × 10^−3^ Å^−1^ which gives *d* being between 0.81 and 1.55 meV. This value is smaller than the DFT calculated DMI 1.88 meV, which can be ascribed to the fact that the Rashba-type DMI was calculated by using only one band close to Fermi level, whereas other bands further from Fermi level may also contribute to the total DMI.

### Electric Field Control of DMI

**Figure 5:**
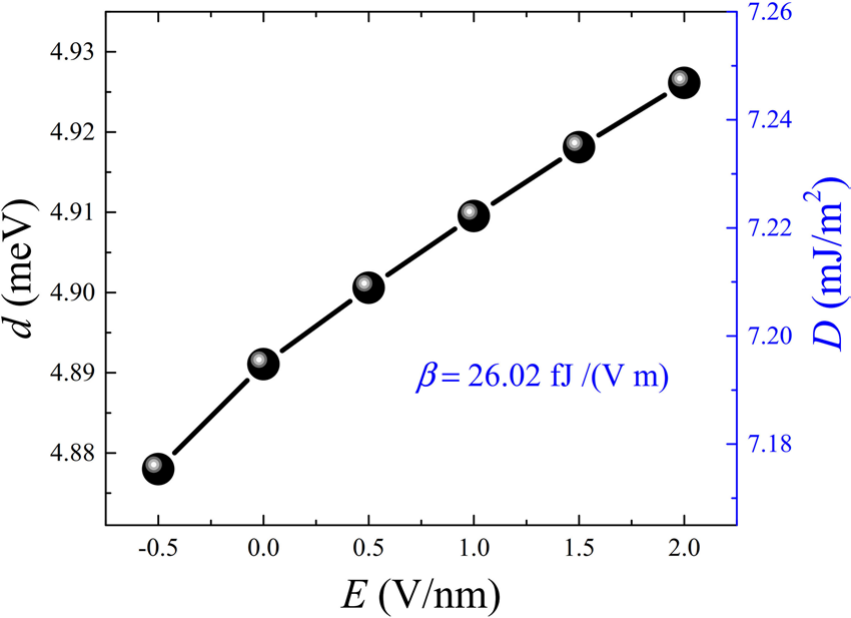
Microscopic and micromagetic DMI for MgO/Co/Pt structure shown in Fig. 4 as a function of electric field applied along normal orientation of the surface. Positive electric field means the electric field is applied from insulator to metal. The slope *β* is indicated for micromagetic DMI.

Finally, we explore the possibility of electric field control of DMI in Oxide/FM/NM structures, or voltage-controlled DMI (VCDMI). Electric field control of interfacial magnetism has recently attracted a very large attention^51–54^, as it provides an additional degree of freedom to manipulate magnetization using gate electric field. While most studies adressed electric field controlled of PMA (VCMA), there is no first-principles study of electric field control of DMI even though it has been experimentally reported recently for the very small DMI case in MgO/Fe/Au multilayer with small DMI^55^. Here we study the effect of an electric field on the DMI in MgO/Co/Pt trilayers. We used 3 ML of Co structure to calculate DMI as a function of external electric field *E* (see Fig. 5). The electric field is applied perpendicularly to the plane of the interface with positive voltage pointing from insulator to metal. It is shown that both microscopic DMI *d* and micromagnetic *D*, are increasing approximately linearly as a function of *E* [Fig. 5]. Similarly to the electric field control of the PMA, the efficiency of the EF control of the DMI (or VCDMI efficiency) can be characterized by the slope of the curve *β* defined as a ratio of the DMI change to *E*, which is found to be equal to 26.02 fJ/(Vm). Interestingly, this parameter is comparable to the slope in electric field control of PMA for Fe(Co)/MgO structures^54–57^. This unveils the possibility of simultaneous tuning of both PMA and DMI within the same range using gate electric field suggesting a route towards an efficient way for controlling magnetic skyrmions since their size and stabilizy depend on both DMI and PMA.

## Conclusion

In conclusion, we proposed three approaches for the efficient tuning of Dzyaloshinskii-Moriya interaction (DMI). The first one is to use NM/FM/Pt trilayers with inverse stacking of FM/Pt and FM/NM structures characterized by DMI with opposite chiralities. This allows the enhancement of DMI up to 50% as compared to the corresponding FM/Pt bilayers. Moreover, we demonstrated that in case of Ir/Fe/Co/Pt multilayers a giant DMI values up to 5.5 meV/atom can be achieved, which is almost twice of that for Co/Pt bilayers. The second approach is to cap Co/Pt structure with an oxidized layer, which can cause a dramatic DMI enhancement due to the Rashba type DMI. Finally, we demonstrated that DMI can be controlled by the application of an electric field in MgO/Co/Pt structure and showed that its efficiency is comparable to E-field control for PMA. These three very efficient approaches pave the way for engineering giant DMI for spintronic applications.
